# Self‐assembled silk fibroin cross‐linked with genipin supplements microbial carbonate precipitation in building material

**DOI:** 10.1111/1758-2229.13202

**Published:** 2023-10-09

**Authors:** Jiayu Li, Varenyam Achal

**Affiliations:** ^1^ Department of Environmental Science and Engineering Guangdong Technion—Israel Institute of Technology Shantou Guangdong China; ^2^ Guangdong Provincial Key Laboratory of Materials and Technologies for Energy Conversion Guangdong Technion—Israel Institute of Technology Shantou Guangdong China

## Abstract

The process of microbially induced carbonate precipitation (MICP) is known to effectively improve engineering properties of building materials and so does silk fibroin (SF). Thus, in this study, an attempt was taken to see the improvement in sand, that is, basic building material coupled with MICP and SF. Urease producing *Bacillus megaterium* was utilized for MICP in Nutri‐Calci medium. To improve the strength of SF itself in bacterial solution, it was cross‐linked with genipin at the optimized concentration of 3.12 mg/mL. The Fourier transform infrared (FTIR) spectra confirmed the crosslinking of SF with genipin in bacterial solution. In order to understand how such cross‐linking can improve engineering properties, sand moulds of 50 mm^3^ dimension were prepared that resulted in 35% and 55% more compressive strength than the one prepared with bacterial solution with SF and bacterial solution only, respectively with higher calcite content in former one. The FTIR, SEM, x‐ray powder diffraction spectrometry and x‐ray photoelectron spectroscopy analyses confirmed higher biomineral precipitation in bacterial solution coupled with genipin cross‐linked SF. As the process of MICP is proven to replace cement partially from concrete without negatively influence mechanical properties, SF cross‐linked with genipin can provide additional significance in developing low‐carbon cement‐based composites.

## Graphical abstract

Silk fibroin (SF) cross‐linked with genipin in bacterial solution facilitated microbially induced carbonate precipitation (MICP), with SF's amino acids enhancing calcification in sand. This SF‐genipin‐MICP combination exhibited superior calcite precipitation compared to bacteria alone. The study suggests that SF cross‐linked with genipin in conjunction with MICP holds promise for developing low‐carbon cement‐based composites.

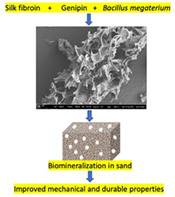

## INTRODUCTION

Microbially induced carbonate precipitation (MICP), a type of biomineralization involving bacterial hydrolysis of urea to carbonate and ammonium ions, has been studied widely in recent years. Various biological processes can lead to MICP, such as ureolysis (Bachmeier et al., 2002), denitrification (O'Donnell et al., 2019), sulphate reduction (Le Pape et al., 2017) and iron reduction (Zeng & Tice, 2014). Among these, the high energy efficiency, low cost, controllable reaction process and direct separation and harvest procedure have made the enzymatic hydrolysis process of urea a highly attractive option that has gained widespread attention (DeJong et al., 2010).

The mechanism of MICP involves the microbial metabolism of organic metabolites such as urea by the action of urease, which is secreted by various microbes, specifically known as ureolytic bacteria including *Sporosarcina pasterurii*, *S. ureae*, *Bacillus megaterium*, *B. cereus*, *B. cohnii*, *B. licheniformis* and *Lysinibacillus sphaericus* among others (De Muynck et al., 2010). This process involves the hydrolysis of urea into ammonia and carbonic acid during the initial stage of the reaction. Subsequently, these compounds react in water to produce bicarbonate, ammonium and hydroxide ions via equilibration. The hydroxide ions lead to raising the pH, which can shift the bicarbonate equilibrium, resulting into ions to precipitate out of solution and form calcium carbonate ions. As a result of this shift, the metal ions can be triggered. The increase in ammonium ions in the vicinity raises the pH level, leading to the autonomous progression of the reaction towards the formation/precipitation of calcium carbonate. The chemical reactions are shown below (Achal & Pan, 2011; Ferris et al., 1996; Li et al., 2022; Mitchell & Ferris, 2005).

(1) $$\mathrm{CO}{\left(NH_{2}\right)}_{2}+2H_{2}O\to 2NH_{3}+H_{2}CO_{3}$$

(2) $$\begin{array}{c} NH_{3}+H_{2}O\to NH_{4}^{-}+OH^{-} \end{array}$$

(3) $$\begin{array}{c} H_{2}CO_{3}\to 2H^{+}+CO_{3}^{2-} \end{array}$$

(4) $$\begin{array}{c} Ca^{2+}+CO_{3}^{2-}=\mathrm{CaC}O_{3} \end{array}$$

The precipitation of calcium carbonate takes place at the bacterial surface in the presence of sufficient concentration of calcium and carbonate ions in the solution.

$$Ca^{2+}+\text{Bacterial cell}\to \text{Cell}‐Ca^{2+}$$

$$\text{Cell}‐{\mathrm{Ca}}^{2+}‐{\mathrm{CO}}_{3}^{2-}\to \text{Cell}‐\mathrm{CaC}O_{3}$$

In the last decade, the number of published papers related to the technical mechanism and applications of MICP has maintained an increasing trend (Song et al., 2022). The application of MICP can be seen in heavy metal immobilization, self‐healing concrete and geotechnical engineering including soil improvement, mitigate seashore erosion and slope failure (Cheng et al., 2021; Fujita et al., 2010; Ivanov & Chu, 2008; Martinez et al., 2021; Ramakrishnan et al., 2001; Yu et al., 2022; Yu & Zhang, 2023). In recent years, the MICP technique has also been introduced for cementing geological formations such as soils and fractured rocks by using biofluids and chemical solutions to induce carbonate precipitation, thereby cementing geological formations (Wang et al., 2022).

Sand is the most important and basic construction material to start with any significant finding in related research on MICP. The precipitation of calcium carbonate in pores between sand particles leads to interparticle binding and biocementation, improves the mechanical properties of sand columns and reduces permeability, which is an important factor in determining the durability of building materials. Furthermore, biocemented sands have recently been enhanced with synthetic fibres, including basalt fibres, carbon fibres and jute fibres (Spencer et al., 2020; Xiao et al., 2019; Yao et al., 2021; Zhao et al., 2020). However, there are some challenges associated with using MICP in building materials. One challenge is the use of urea as a substrate. Urea is a common substrate for MICP, but it causes environmental injustice by releasing high amounts of ammonia into the atmosphere. Another challenge is the use of synthetic fibres. Most of the fibres that have been studied for use in MICP are either chemically synthesized or originate from plants. These fibres can be expensive and difficult to obtain. On the other hand, the addition of inorganic components, such as calcium carbonate, to organic polymers noticeably improves the mechanical, barrier and thermal properties of composite materials (Messersmith & Giannelis, 1994; Ray et al., 2002) and this concept can be achieved by coupling precipitation reactions with self‐organizing or self‐assembled proteins such as silk fibroin (SF). SF is a natural protein that can be used to create fibres with excellent mechanical and barrier properties. When SF is combined with CaCO_3_, it forms a composite material that is strong, durable and resistant to environmental degradation.

SF, isolated from *Bombyx mori*, is a natural organic polymer that displays excellent biological and mechanical properties (Bhattacharya et al., 2017). It is being investigated for applications in the field of tissue engineering in blood vessels, skin, bone and cartilage (Kim & Park, 2016). Here, we show a new approach for biomineralization in sand mediated by a ureolytic bacterium in the presence of SF. Ureolytic bacteria induce the precipitation of calcium carbonate, while SF acts as an organic polymer to enhance the overall mechanical properties of the produced sandstone. However, the random coil conformations of macromolecules, which are dominant in aqueous solutions of SF, reduce its strength (Zhang & Pan, 2019). Cross‐linking of SF via chemical methods is a time‐saving strategy to improve the strength of SF. However, most chemicals used as cross‐linking agents have toxic effects on bacterial cells. Thus, in this study, genipin was used to promote the formation of β‐sheets in SF (Silva, Maniglio, et al., 2008; Silva, Motta, et al., 2008). It is noteworthy that genipin, derived from gardenia fruit, is much less toxic than glutaraldehyde and many other commonly used synthetic cross‐linking reagents.

The objective of this study was to develop a new method for creating sustainable building materials. The method involves cross‐linking SF with genipin in a nutrient solution, followed by coupling of microbial calcium carbonate precipitation with the cross‐linked SF in sand. The present study focused on utilizing *B. megaterium* in inducing carbonate precipitation in sand supported by genipin cross‐linked SF to assist in improving the mechanical strength, followed by elucidating the mechanism of the process.

## EXPERIMENTAL PROCEDURES

### Materials

The well‐known ureolytic bacterium *B. megaterium* CGMCC 1.1741 was used in this study. In widely researched nutrient broth urea media (Li et al., 2021), the bacterium showed urease activity of 44 U/mL. The bacterial strain was maintained on nutrient agar medium (pH 8.0). To induce calcium carbonate precipitation and support bacterial growth in sand, Nutri‐Calci medium with the following composition (per L): 5 g tryptone, 5 g yeast extract, 2 g NaCl and 40 mM CaCl_2_ (pH 7.5) was used throughout the study.

Quartz sand, locally collected, was used as an aggregate in the present study with grain size characteristics d10 = 150 μm (10% of the grains were smaller than this diameter) and d90 = 300 μm.

### Cell viability assay and crosslinking of silk fibroin with genipin

Although genipin is known to have low acute toxicity, with an LD_50_ i.v. 382 mg/kg in mice, it was important to test its toxicity on bacterial cells. Thus, before the cross‐linking reaction of SF with genipin, the toxicity of genipin on *B*. *megaterium* growth in nutrient broth medium was determined via a microdilution protocol using a plate reader. The minimal inhibitory concentration (MIC) of genipin was determined in duplicate columns of a 96‐well plate, following Brouwers et al. (2020), with each well containing 190 μL of nutrient broth supplemented with genipin. A stock solution of genipin was prepared by dissolving it in dimethyl sulfoxide to a final concentration of 25 mg/mL (Koudouna et al., 2021). The top row contained the highest concentration of genipin, and the genipin concentration was diluted down the column, with the bottom well containing no genipin. Each well was then inoculated with 10 μL of *B*. *megaterium* that was growing exponentially at an OD600 of approximately 0.2–0.3. After inoculation, the plate was incubated for 24 h at 30°C with orbital shaking at 600 rpm (in the plate reader). The bacterial growth was determined by visual inspection of the turbidity of the wells after incubation, followed by OD600 measurement. In each plate, at least one column contained only growth medium as a control for contamination.

The MIC of genipin against *B*. *megaterium* shown by no growth as measured using a plate reader was 6.25 mg/mL. Although genipin is known to have less inhibitory bactericidal effect, its toxicity cannot be ignored, as it will affect the process of carbonate precipitation due to poor bacterial growth. An earlier study reported that bacteria including *S. aureus* and *P. aeruginosa* were sensitive to genipin at concentrations of 3.12 and 1.56 mg/mL, respectively (Koudouna et al., 2021).

To crosslink the genipin inside the fibroin solution, genipin at nonlethal concentrations to bacterial cells was added to the SF solution (0%, 1%, 2% and 4% m/v), stirred for a few minutes and then finally added to bacterial culture grown in Nutri‐Calci medium (OD600 = 1) at 30°C to allow the crosslinking reaction to take place. A change from the transparent yellowish colour to violet opaque was observed from 0 to 24 h, indicating a successful crosslinking reaction. The solution was lyophilized, followed by evaluation of secondary structure changes using an attenuated total reflectance Fourier transform infrared (FTIR) spectrophotometer.

### Sand mould specimen preparation

The coupling of microbial calcium carbonate precipitation with genipin cross‐linked SF was studied in sand. This was done by preparing a sand mould with dimensions of 50 mm^3^. Sand was added to the mould in layers, and after each layer, the optimized concentration of SF solution with genipin and bacterial culture grown in Nutri‐Calci medium (OD_600_ = 1) were incubated for 24 h to proceed with the cross‐linking reaction. The top layer of the sand mould was sprayed with a sufficient amount of the same solution to ensure complete saturation of the sand. Control specimens were treated with (a) distilled water, (b) Nutri‐Calci medium and (c) bacterial culture grown in Nutri‐Calci medium (OD_600_ = 1). All the specimens were cured for 7 days and sprayed with Nutri‐Calci medium as a protein source for bacterial growth and a calcium source for biomineralization at an interval of 24 h. The experiments were performed at 37°C in triplicate. At the end of 7 days, the specimens were removed from the moulds and air‐dried for at least 48 h before further analyses.

### Unconfined compressive strength, calcite mass estimation and porosity

The sand specimens were subjected to unconfined compressive strength (UCS) testing. Calcite contents from sand specimens were measured by treating them with HCl (5 mol/L) and calculated accordingly using a gravimetric acid washing technique (Mortensen et al., 2011). Briefly, the oven‐dried mass of the sand specimen was measured before and after an acid wash (5 mol/L HCl). The dissolved calcium carbonate–acid wash solution was rinsed multiple times through a 200‐mesh sieve, allowing the dissolved salts to be rinsed from the specimen.

Calcite content (%) = 𝑚𝐶𝑎𝐶𝑂_3_/𝑚𝑠𝑝𝑒𝑐𝑖𝑚𝑒𝑛 × 100%, where, 𝑚𝐶𝑎𝐶𝑂_3_ and 𝑚𝑠𝑝𝑒𝑐𝑖𝑚𝑒𝑛 are the calcite mass and dry mass of specimen, respectively.

The porosity and pore size distribution of sand specimens were determined using mercury porosimetry (Quantachrome PoreMaster 33 porosimeter), with a radius range of 0.0064–950 μm (Fang et al., 2021). All specimens were dried at 50°C for 24 h prior to porosity testing.

### Microstructural analyses

The morphologies of microstructures inside sand specimens were observed through scanning electron microscopy, while functional groups associated with biomineralization reactions coupled with cross‐linked SF were analysed under FTIR. SEM analysis was performed to analyse the surface topography, morphology and mineralogical composition of the precipitate in all samples using a scanning electron microscope. The biominerals were identified by x‐ray powder diffraction spectrometry (XRD) analyses with the PDF‐2 database of the International Center for Diffraction Data.

### X‐ray photoelectron spectroscopy

X‐ray photoelectron spectroscopy (XPS) experiments were further conducted, to confirm the precipitation of calcium carbonate in sand specimens, using PHI5000 VersaProbe III Photoelectron Spectrometer (Japan). Al Kα x‐rays were used as the excitation source (*hv* = 1486.6 eV). Binding energies (BEs) of the samples were referenced using the C(1s) peak of adventitious carbon at 285 eV and measured with an accuracy of ±0.2 eV. High‐resolution C(1s), Ca(2p) and O(1s) spectra were obtained at a pass energy of 50 eV. The XPS data were acquired at a takeoff angle of 55°. Furthermore, multiple component XPS peaks were defined by a peak fitting program that assumed a 100% Gaussian peak shape.

## RESULTS AND DISCUSSION

### Evaluation of SF crosslinking

**FIGURE 1:**
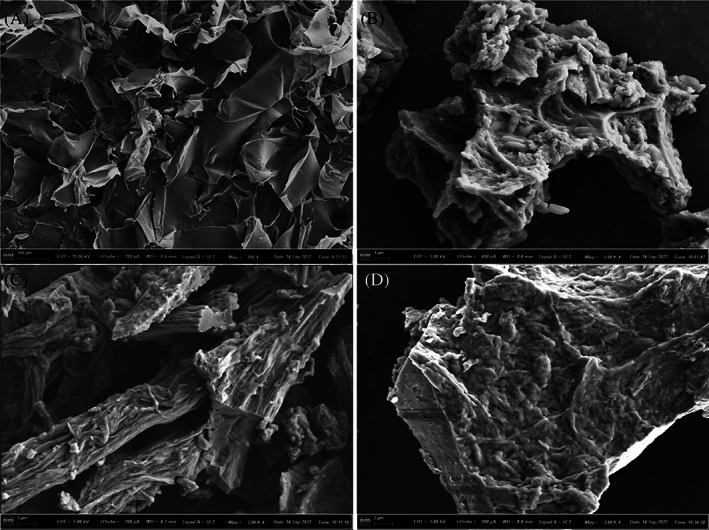
Scanning electron micrography of (A) raw SF, and bacterial cells adhered on the surface of (B) 1% SF, (C) 2% SF and (D) 4% SF. A typical image is shown from many similar examples.

For crosslinking, genipin at the concentration of 3.12 mg/mL was added into Nutri‐Calci medium amended with SF and bacterium. The crosslinking of SF with genipin was demonstrated by the change in colour from golden and transparent to dark opaque violet (Figure S1A–C). The result was in agreement with Bucciarelli et al. (2021). These samples were further observed under SEM. Raw SF showed a stable, uniform and smooth surface under SEM (Figure 1A,B). Such a structure is SF, which is known to promote cell adhesion and proliferation (Chlapanidas et al., 2011). Such structures posses a network of interconnected pores, an arrangement that is similar to that of porous structure scaffolds (Wang et al., 2019).

Bacterial cells were found to adhere on the surface of SF; however, this adhesion was more in an organized frame as bundle fibre with crystals probably of carbonates (confirmed later with XRD) when SF at the concentration of 2% (Figure 1C) was used compared to 1% (Figure 3) or 4% (Figure 1D). At 4% concentration, the SF may have been too dense and the bacterial cells may have had difficulty adhering to the surface. The presence of carbonate crystals at 2% concentration may have provided a more favourable environment for the bacterial cells to grow. Further, the carbonate crystals may have provided a surface for the bacterial cells to attach to and may have also provided nutrients that the bacterial cells needed to grow. The 2% concentration may have simply been the optimal concentration for bacterial adhesion. At this concentration, the SF provided the right amount of surface area for the bacterial cells to adhere to without being too dense, where the presence of carbonate crystals may provide a more favourable environment for the bacterial cells to grow.

**FIGURE 2:**
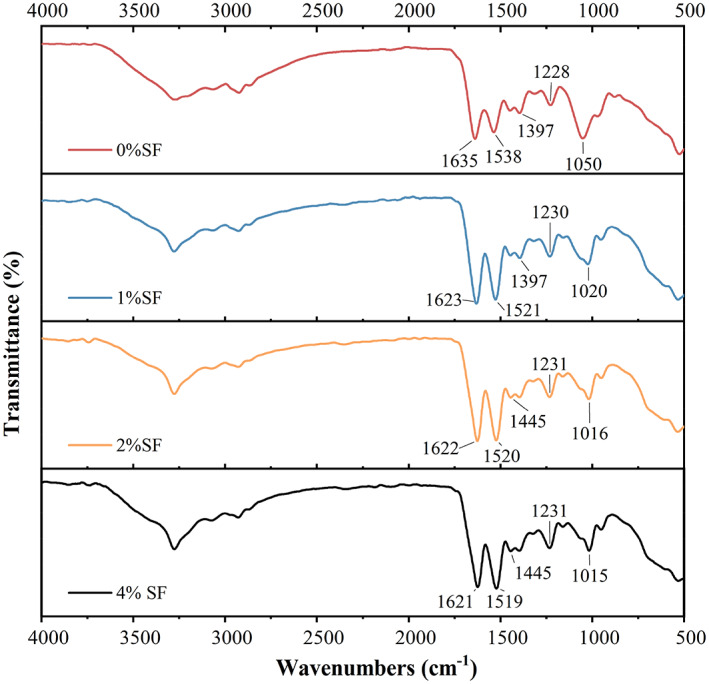
FTIR spectroscopy of precipitate obtained from bacterial cells grown in Nutri‐Calci medium in various samples cross‐linked with genipin. Typical data are shown from one of several determinations.

All the samples of SF at different concentrations in bacterial culture grown in Nutri‐Calci medium with genipin at a concentration of 3.12 mg/mL were lyophilized and evaluated for crosslinking using FTIR (Figure 2). The raw SF has three different conformations: random coil, Silk I (α‐form) and Silk II (β‐sheet). The hypothesis that was tested is that the structure of SF molecules could be rearranged by the presence of genipin, together with the bacteria and effect of biomineralization.

The adsorption bands of amide I migrated from 1635 cm^−1^ in the 0% SF sample to approximately 1622 cm^−1^ in the 1%, 2% and 4% (m/v) SF samples with genipin. This shift indicates that the hydrogen bonds between the C=O and NH_2_ groups in the amide bonds of SF were broken by genipin, releasing free C=O groups (Zeng et al., 2015). The breaking of these hydrogen bonds is likely due to the formation of covalent bonds between the genipin molecule and the C=O and NH_2_ groups in the amide bonds of SF. These covalent bonds prevent the C=O and NH_2_ groups from forming hydrogen bonds with each other, which causes the amide I band to shift to a lower wavenumber.

The characteristic band at 1411 cm^−1^ shifted to a higher wavelength of 1446 cm^−1^, which may show that the ring stretching vibrations mixed strongly with —CH in‐plane bending. The adsorption bands at 1050 cm^−1^ shifted to 1020, 1016 and 1015 cm^−1^ in the 1%, 2% and 4% SF samples, respectively, after the addition of genipin, owing to the reaction of —NH_2_ with genipin (Wang et al., 2013). These results demonstrated that genipin changed the conformation of SF as a result of structural rearrangement of chains to form covalent bonds. Genipin and *B. megaterium* are important chemical cross‐linkers that induce SF molecular formation from random coils and α‐helices to β‐sheets (Silva, Maniglio, et al., 2008; Silva, Motta, et al., 2008; Wang et al., 2013).

Overall, the changes in the infrared spectra of SF with the addition of genipin provide evidence that genipin interacts with SF and breaks the hydrogen bonds between the C=O and NH_2_ groups in the amide bond. This interaction also causes the aromatic ring of tyrosine to interact with the genipin molecule and the N—H group to react with the genipin molecule. The interaction of genipin with SF can be used to modify the properties of SF. For example, genipin can be used to cross‐link SF, which makes it stronger and more durable. Genipin can also be used to immobilize proteins on SF, which makes it possible to use SF as a biomaterial.

Amide II (1520–1530 cm^−1^) was not observed in samples without SF (Figure 4) but in SF at concentrations of 0%, 1%, 2% and 4% (m/v) crosslinked with genipin due to the conformation change from random coils or α‐helices to β‐sheets (Zhang et al., 2010). The intense absorption peaks at approximately 1520 and 1230 cm^−1^ are characteristic absorption peaks of β‐sheets (Figure 2). The spectrum of samples of genipin with SF showed a strong absorption peak at approximately 1622 cm^−1^ that was attributed to the C=C vibration of the olefin ring in genipin during the cross‐linking reaction (Li et al., 2015). In the case of cross‐linking SF by genipin, some minor variations in the peak position and intensity were observed. For the crosslinking mixture, the peak at 1622 cm^−1^ was more intense in SF at a concentration of 2%, and it did not appear in the absence of SF in the sample (Figure 2). The increase in the intensity of the peak at 1622 cm^−1^ suggests that the cross‐linking reaction between SF and genipin was more effective at this concentration, thus, 2% (m/v) SF was chosen for all other experiments. The appearance of a band at approximately 1170 cm^−1^ could be due to the cross‐linking reaction between the amino acids present in SF and genipin (Dimida et al., 2017). Genipin can form monomer polymerization with amino acids present in SF through the formation of covalent bonds between the aldehyde group and the secondary amine and the formation of double linkages in the carbon ortho‐position (Wang et al., 2013).

### Unconfined compressive strength and calcite in sand specimens

**FIGURE 3:**
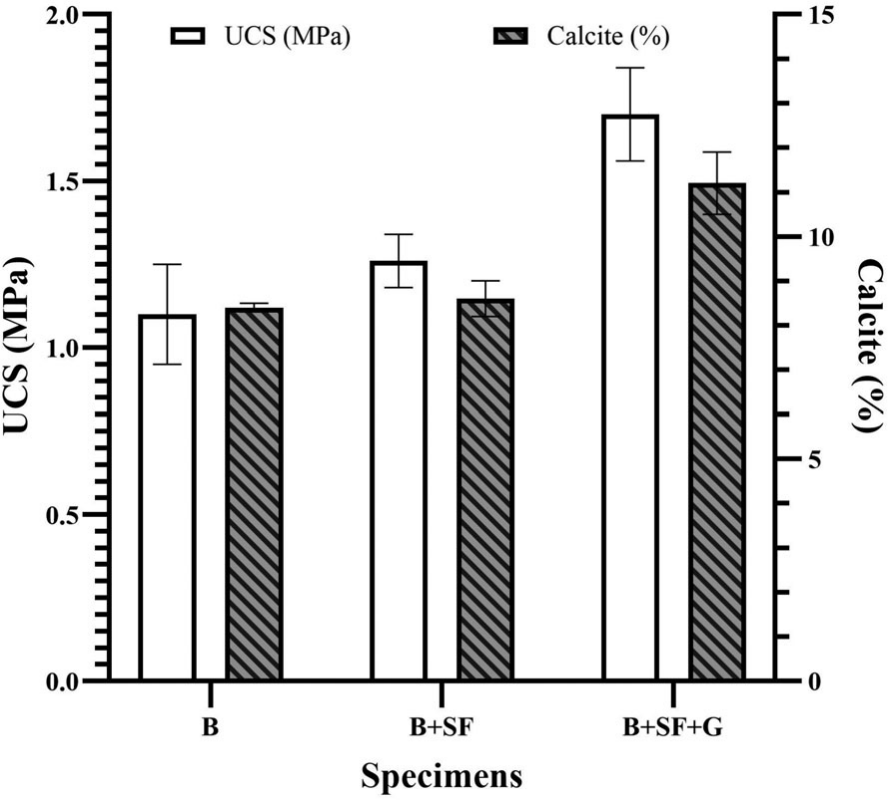
UCS of sand specimens prepared with B (bacteria), B + SF (bacterial cells with 2% (m/v) SF) and B + SF + G (bacterial cells with 2% (m/v) SF cross‐linked with genipin), and calcite content in each specimen.

There was obvious variation in the UCS of sand specimens prepared with the cross‐linking reaction compared to specimens prepared with 2% (m/v) SF and with or without bacterial cells grown in Nutri‐Calci medium. Furthermore, the results were in accordance with the calcite contents in the respective specimens, and sand moulds had higher calcite contents. Control specimens made only with Nutri‐Calci medium disintegrated due to the lack of any biochemical reaction in the absence of bacterial cells. On the other hand, a UCS of 1.1 MPa was obtained in sand cubes prepared with a culture of bacterial cells grown in Nutri‐Calci medium (Figure 3). The significant improvement in terms of UCS was measured in sand cubes prepared in bacterial culture with 2% (m/v) SF cross‐linked with genipin (1.7 MPa), contrary to the same without genipin (1.26 MPa). Although there could not be a direct comparison of UCS results with other reports (Chen et al., 2022; Shan et al., 2022; Yao et al., 2021), it is clear that biomineralization with genipin cross‐linked SF significantly improved the UCS of sand.

Additionally, the UCS of the sand specimens increased with an increase in calcium carbonate content. The calcite content in sand cubes prepared with bacterial cells grown in Nutri‐Calci medium and with 2% (m/v) SF was 8.4% and 8.6%, respectively; however, a significant improvement in calcite percentage (11.2%) was observed in sand cubes prepared in bacterial culture with 2% (m/v) SF cross‐linked with genipin (Figure 3). The findings are in agreement with Rong and Qian (2012), according to which a calcite content higher than 8% brings a significant increase in compressive strength in sand stone due to biomineralization.

The compressive strength of concrete‐type specimens decreases gradually with increasing porosity and pore size, and this warrants porosity analysis (Zhang et al., 2020). Precipitation of minerals like calcium carbonate is an effective way to reduce porosity (Beckingham, 2017; Chagneau et al., 2015). This is evident in Table 1, which shows that both porosity and pore size were further reduced in specimens prepared in bacterial culture with 2% (m/v) SF cross‐linked with genipin. The results were consistent with the compressive strength and calcite content. This further confirms why MICP is a promising geotechnique to seal leakage pathways in the subsurface or to stabilize soils or in other construction engineering.

**TABLE 1:** Porosity analysis of various sand specimens.

Parameter	Specimens		
	B	B + SF	B + SF + G
Total pore area (m^2^/g)	6.40 (±0.33)	6.00 (±0.25)	5.60 (±0.26)
Total intrusion volume (mL/g)	0.11 (±0.00)	0.09 (±0.00)	0.05 (±0.00)
Volume‐median pore size (nm)	82.40 (±0.47)	84.60 (±0.52)	89.50 (±0.44)
Average pore size (nm)	60 (±0.51)	57 (±0.62)	53 (±0.58)
Porosity (%)	17.20 (±0.78)	16.40 (±0.91)	14.20 (±0.81)

**FIGURE 4:**
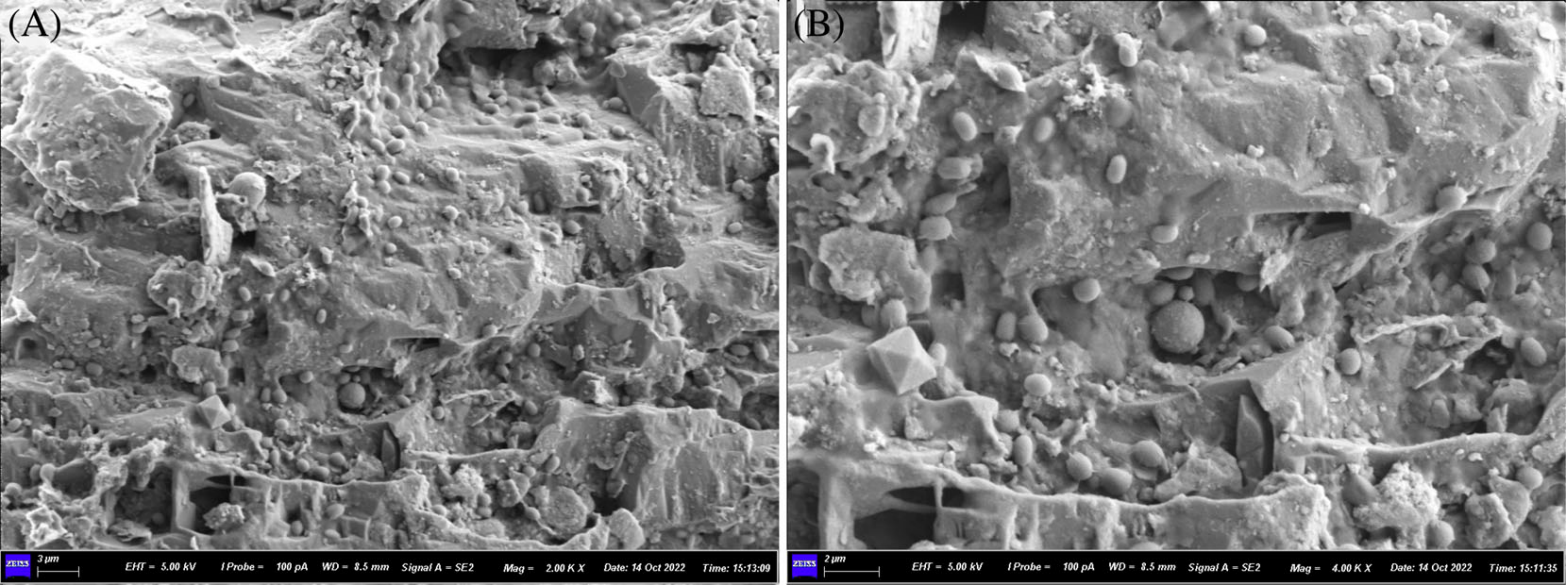
SEM in sand specimens containing 2% (m/v) SF cross‐linked with genipin showing (A) bacterial cells embedded on fibrous structure along with biominerals and (B) closer view of (A). A typical image is shown from many similar examples.

**FIGURE 5:**
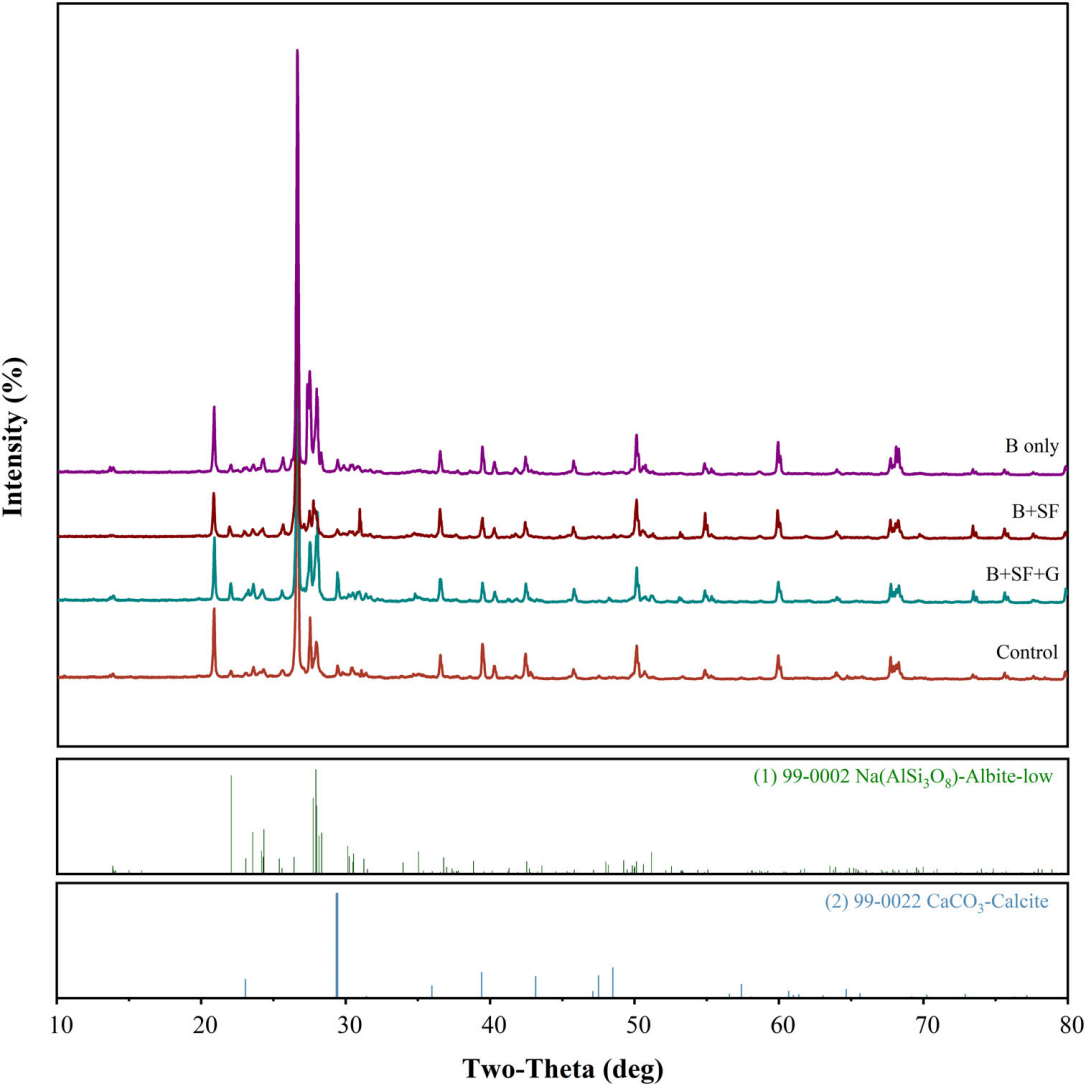
X‐ray diffraction of minerals formed in sand specimens with bacteria (B only), bacteria with 2% (m/v) SF (B + SF) and bacteria grown in the presence of 2% (m/v) SF cross‐linked with genipin (B + SF + G), compared to control (Nutri‐Calci medium). Typical data are shown from one of several determinations.

The arrangement of bacterial cells embedded on the fibrous structure along with several crystals was observed under SEM in sand specimens containing 2% (m/v) SF cross‐linked with genipin (Figure 4). Based on this information, it can be concluded that the presence of SF and genipin have acted as a scaffold or template for the accumulation of microbial calcite in these sand specimens. The biomineralization process has been enhanced, likely due to the presence of the SF and/or genipin which have created a favourable environment for the microbial cells to interact with and accumulate calcium carbonate. The SEM images show the embedded bacterial cells and crystal structures, suggesting that the biomineralization process is complete and successful. The crystals/minerals formed due to biomineralization were confirmed as calcite (CaCO_3_) with a prominent stronger peak identified under XRD in specimens again with 2% (m/v) SF cross‐linked with genipin (Figure 5). In the context of identifying calcite in sand, XRD has been found to be a reliable method for differentiating between grains of calcite and quartz. Studies have shown that the characteristic reflection patterns that arise from XRD analysis allow for reliable identification of calcite, as quartz grains display significantly different XRD patterns (Hupp & Donovan, 2018). It is a well‐known phenomenon that calcite plugs pores between sand particles and improves the mechanical properties of the resulting sand cubes (Huang et al., 2016; Wang & Liu, 2021; Whitaker et al., 2018).

### XPS

A typical XPS wide scan spectrum from all sand specimens prepared using 2% (m/v) SF cross‐linked with genipin, without genipin, and with bacterial cells only grown in Nutri‐Calci medium as compared to control calcite is shown in Figure S2 where all three elements comprising CaCO_3_, that is, Ca, C and O, were observed. The intensity of Ca(2s) was significantly higher in sand specimen containing 2% (m/v) SF cross‐linked with genipin, followed by same but without genipin. The surface elementary compositions with atomic concentration of C(1s), O(1s) and Ca(2p) for all specimens are summarized in Table 2. The concentration of Ca(2p) in specimens can be seen in increasing order from control to bacteria only, followed by bacteria with SF and SF cross‐linked with genipin supported by bacteria. All specimens exhibited elevated levels of carbon due to the high surface energy of the calcium carbonate surfaces (Ni & Ratner, 2008). Data were analysed by the CasaXPS software (Fairley et al., 2021). The results suggest that the addition of SF cross‐linked genipin can significantly accelerate the MICP process in the environment. This could potentially enhance the capability of MICP to be used in heavy metal bioremediation and biocementation.

**TABLE 2:** Atomic concentration table of the XPS measurement on C (1s), O (1s) and Ca (2p).

Groups	C (1s)	O (1s)	Ca (2p)
	At%^a^	At%	At%
Control	59.46	39.15	1.39
Only bacteria	55.09	42.11	2.80
Bacteria + SF	51.10	45.84	3.05

^a^ Percentage atomic concentration.

**FIGURE 6:**
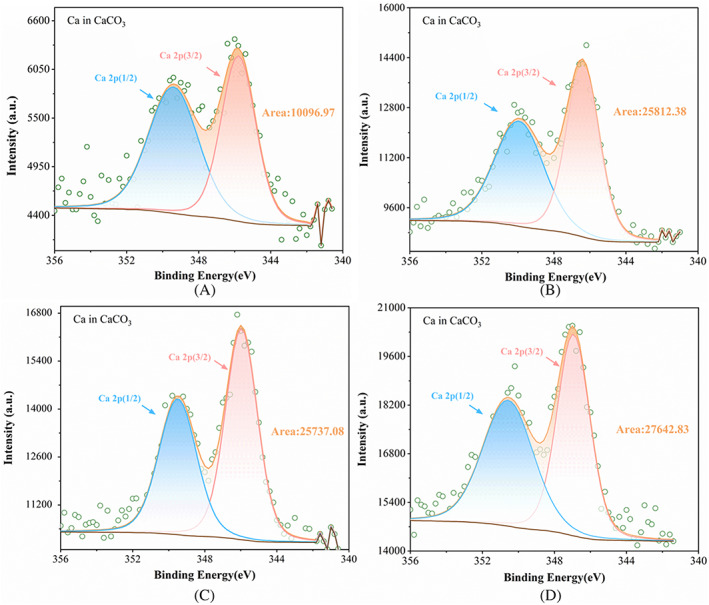
XPS Ca2p core level spectra as observed in various sand specimens with (A) control, (B) bacteria only, (C) bacteria with 2% (m/v) SF and (D) bacteria grown in the presence of 2% (m/v) SF cross‐linked with genipin.

The high‐resolution XPS spectra of the Ca2p core levels from all sand specimens is presented in Figure 6A–D where two peaks were seen, identified as Ca2p1/2 and Ca2p3/2, in the order of increasing BE. All the specimens presented a strong and sharp peak in the Ca2p1/2 region, while displayed much stronger peak in the Ca2p3/2 region of high intensity but these peaks were of much lower intensity in the control sand specimens. The results were in agreement with Rowley et al. (2021). The position of the most intense Ca2p3/2 peak is found to be strongly dependent on the local chemical environment of the Ca atom (Brigiano et al., 2022). The Ca2p3/2 core level BE position of calcite is known to appear at 346.5 (Ni & Ratner, 2008) that confirms significantly higher precipitation of calcite in sand specimens prepared using 2% (m/v) SF cross‐linked with genipin compared to other specimens.

Further, Ca2p3/2‐2p1/2 splitting was observed at 3.55 eV. In the case of calcium carbonate, the Ca2p3/2‐2p1/2 splitting of 3.55 eV is a characteristic of the material and can be used to confirm its identity (http://www.xpsfitting.com/2018/05/calcium.html). The results from XPS supported other data from SEM, FTIR and XRD, and confirmed the process of MICP and identified the role of bacteria and SF cross‐linked with genipin in mediated CaCO_3_ precipitation.

### Possible reactions

SF is mainly composed of amino acids, including glycine and alanine, at 46% and 30%, respectively (Zhou et al., 2000). Other amino acids include serine, tyrosine and valine. Due to the high alanine content of SF, the biochemical reactions involved in the biomineralization mechanism can induce carbonate precipitation in sand in the presence of genipin cross‐linked SF. As a result, the following reactions may occur.

$$C_{3}H_{7}{\mathrm{NO}}_{2}+{\text{3O}}_{2}\to {\mathrm{NH}}_{3}+{\text{3CO}}_{2}+{\text{2H}}_{2}O.$$

$${\mathrm{NH}}_{3}+H_{2}O⇌{\mathrm{NH}}^{4+}+{\mathrm{OH}}^{-}.$$

$${\mathrm{CO}}_{2}+H_{2}O⇌H_{2}{\mathrm{CO}}_{3}⇌H^{+}+{\mathrm{HCO}}^{3-}.$$

$${\mathrm{Ca}}^{2+}+{\mathrm{HCO}}^{3-}⇌{\text{CaCO}}_{3}+H^{+}.$$

During the reactions, one mol of alanine produced only one mol of ammonia with three mol of carbon dioxide, which further equilibrated in water, resulting in the formation of ammonium ions that gave rise to an increase in pH, hydroxide ions and bicarbonate ions. Finally, the increased carbonate concentration will induce an increase in supersaturation level leading toCaCO_3_ precipitation around the bacterial cell in the presence of soluble calcium ions (Li et al., 2015; Zhu et al., 2016).

It is noteworthy that the above reactions involving alanine produce less ammonia compared to the ureolytic process, where the hydrolysis of one urea molecule produces two ammonia ions as per the reaction below.

$$\mathrm{CO}{\left({\mathrm{NH}}_{2}\right)}_{2}+H_{2}O\to {\text{2NH}}_{3}+{\mathrm{CO}}_{2}.$$

An earlier study also confirmed the involvement of another amino acid, asparagine, in calcium carbonate precipitation induced by *B. megaterium*, which produced alanine as an intermediate product (Li et al., 2015). It has been reported that amino acids act as templates in the biomineralization of CaCO_3_ (Feng et al., 2022).

## CONCLUSIONS

The process of MICP, a type of biomineralization, is known to enhance the mechanical properties of building materials. On the other hand, SF displays excellent mechanical properties and can positively supplement the MICP process. The research presented in this study revealed that tuning the mechanical and biological properties of SF by cross‐linking with genipin can significantly enhance the biomineralization and compressive strength of sand. The amino acids present in SF can serve as substrates to carry out MICP reactions. The self‐assembled SF molecules cross‐linked with genipin create a network of SF fibres that could be used as a microbial scaffold to promote MICP. Genipin is a natural compound derived from gardenia fruit that acts as an effective cross‐linking agent, and the resulting SF network is biocompatible, allowing for the growth of microorganisms. The microbial scaffold serves as a platform on which the microorganisms can attach and thrive, providing an ideal environment for the production of carbonates. The calcium carbonate produced from the MICP process is then combined with the building material, promoting strength and durability. The results will also have significance in other relevant studies involving MICP, whether in biocementation or heavy metal remediation.

## AUTHOR CONTRIBUTIONS

**Jiayu Li:** Data curation (lead); formal analysis (equal); software (lead); writing – original draft (supporting). **Varenyam Achal:** Conceptualization (lead); methodology (lead); writing – original draft (lead); writing – review and editing (lead).

## CONFLICT OF INTEREST STATEMENT

The authors declare no conflicts of interest.

## Supporting information


**Data S1:** Supporting Information

## Data Availability

The data that support the findings of this study are available from the corresponding author, Varenyam Achal, upon reasonable request.
