# Supplementary material for: Self‐assembled silk fibroin cross‐linked with genipin supplements microbial carbonate precipitation in building material
Source: Environ Microbiol Rep. 2023 Oct 9;15(6):797–808. doi: 10.1111/1758-2229.13202 (PMC10667665; doi:10.1111/1758-2229.13202)
Supplement: Supplementary file 1 — Data S1: Supporting Information [file EMI4-15-797-s001.doc]

**Supplementary Material**

**Self-assembled silk fibroin cross-linked with genipin supplements microbial carbonate precipitation in building material**

**Jiayu Li^1,2^ and Varenyam Achal^1,2*^**

*^1^Department of Environmental Science and Engineering, Guangdong Technion - Israel Institute of Technology, 241 Daxue Road, Shantou, Guangdong, China, 515063*

*^2^Guangdong Provincial Key Laboratory of Materials and Technologies for Energy Conversion, Guangdong Technion - Israel Institute of Technology, 241 Daxue Road, Shantou, Guangdong, China, 515063*

***Corresponding author:** *Department of Environmental Science and Engineering, Guangdong Technion - Israel Institute of Technology, 241 Daxue Road, Shantou, Guangdong, China, 515063.* E-mail: varenyam.achal@gtiit.edu.cn


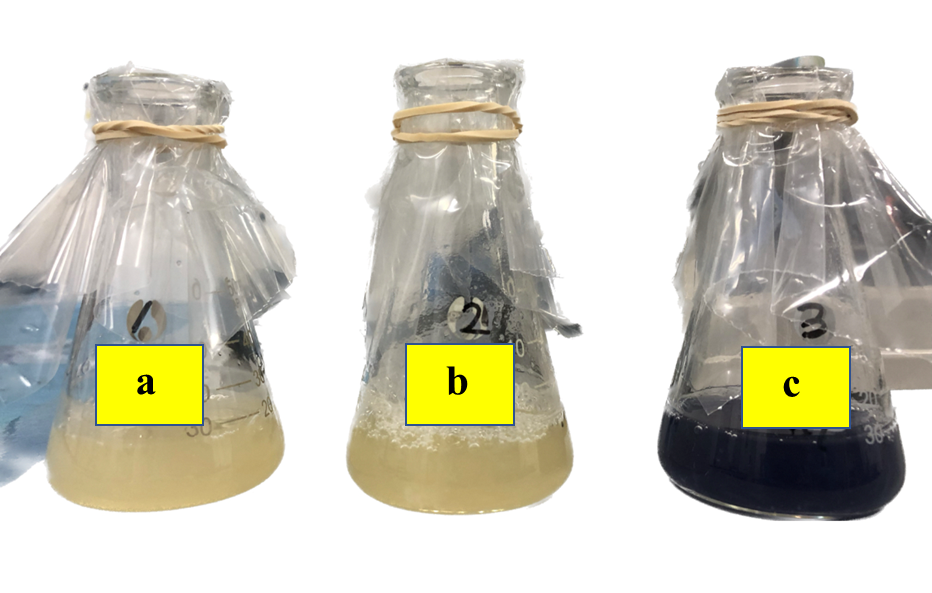


**Fig. S1** Bacterial culture in a.) Nutri-Calci medium, b.) Nutri-Calci medium with silk fibroin, and c.) Nutri-Calci medium with silk fibroin crosslinked by genipin


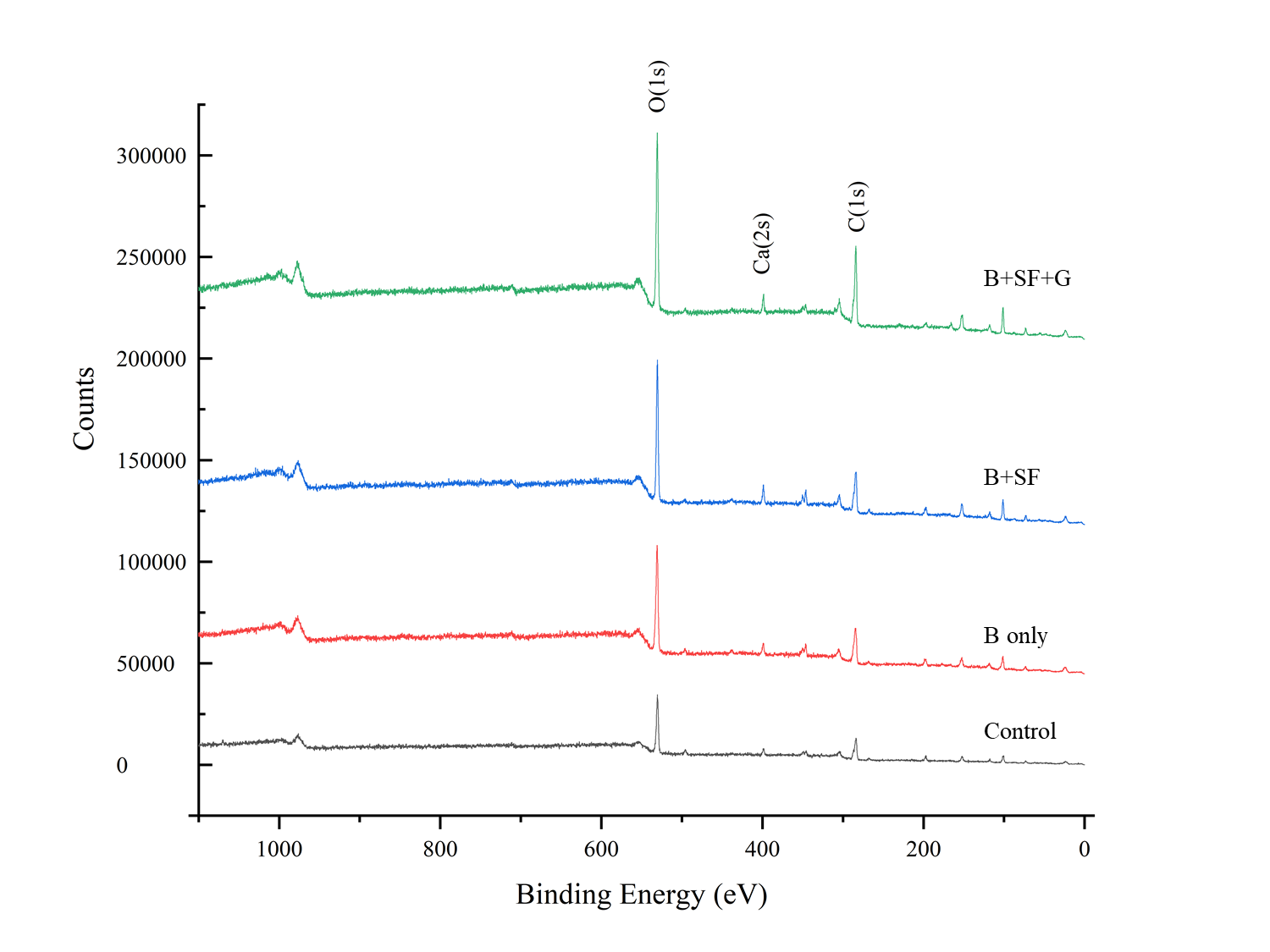


**Fig. S2** XPS wide scan for precipitation in various sand specimens showing that Ca, C, and O, the three compositional elements of calcium carbonate, are present
